# Cholestasis and congenital neuroblastoma in a preterm neonate: a case report

**DOI:** 10.1515/crpm-2021-0089

**Published:** 2022-12-19

**Authors:** Surasak Puvabanditsin, Melissa Guillermo, Yisha Cheng, Olga Sudol, Rajeev Mehta

**Affiliations:** Department of Pediatrics, Rutgers Robert Wood Johnson, Medical School, New Brunswick, NJ, USA; Department of Pediatrics, Rutgers Robert Wood Johnson Medical School, 1 Robert Wood Johnson Place, New Brunswick, NJ 08903, USA

**Keywords:** congenital, neuroblastoma, preterm neonate

## Abstract

**Objectives:**

Neuroblastoma (NB) is one of the most common tumor during perinatal period. The clinical features of NB occurring in fetuses and neonates differ from that in the older age groups. Frequently, Congenital neuroblastomas are incidentally detected prenatally. Clinical presentations of NBs in neonates are highy variable.

**Case presentation:**

A 24-day old preterm 32 weeks’ gestation male neonate developed cholestasis that lead to the diagnosis of stage MS neuroblastoma. There was no NB primary site identified.

**Conclusions:**

To the best of our knowledge, this is the first case report of metastatic NB (Stage MS) in a preterm neonate presenting with cholestsis but without any identifiable adrenal or extra-adrenal primary.

## Introduction

Neuroblastoma (NB) is a tumor of primordial neuroectodermal cells that develops in the embryonic nervous system throughout the fetal and neonatal periods. It is one of the most common neonatal tumors, with an incidence of 0.61 per 100,000 live births [1]. Congenital NBs comprise about 5% of the total annually diagnosed neuroblastomas and usually carry a good outcome despite the metastatic invasion. They carry a normal *MYCN*, neuroblastoma oncogene, copy number and hyperdiploid DNA index [2, 3]. Spontaneous regression of the tumor is likely to be seen in neonates.

Neonatal cholestasis is a disorder of hepatobiliary and metabolic dysfunction in neonates. The incidence is about 1 in 2,500 infant [4]. Neonatal cholestasis has many different causes including viruses, obstructive causes, metabolic diseases, or genetic disorders such as alpha-1-antitrypsin deficiency and Alagille syndrome. The obstructive cause of neonatal cholestasis by a neoplastic invasion of the liver is extremely rare. Our case is unique because it was the cholestasis that lead us to the diagnosis of the malignant neoplasm in this preterm neonate. We report a case of congenital NB that presented as neonatal cholestasis. This case report includes a review of the literature on congenital NBs.

## Case presentation

**Figure 1:**
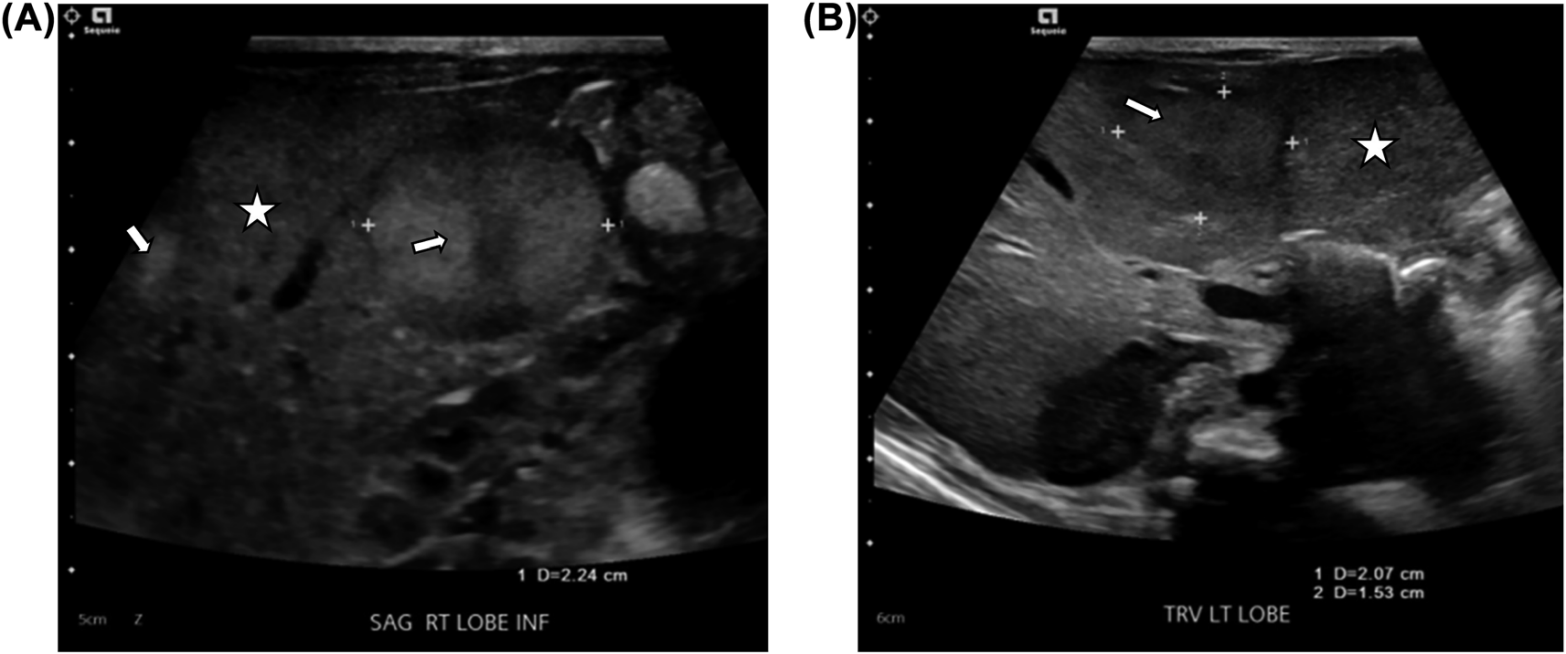
Gray scale sonogram of the liver shows small and large echogenic mass (arrows) on the left and right lobes against the diffusely abnormal hepatic echogenicity (star) background of normal hepatic echotexture.

**Figure 2:**
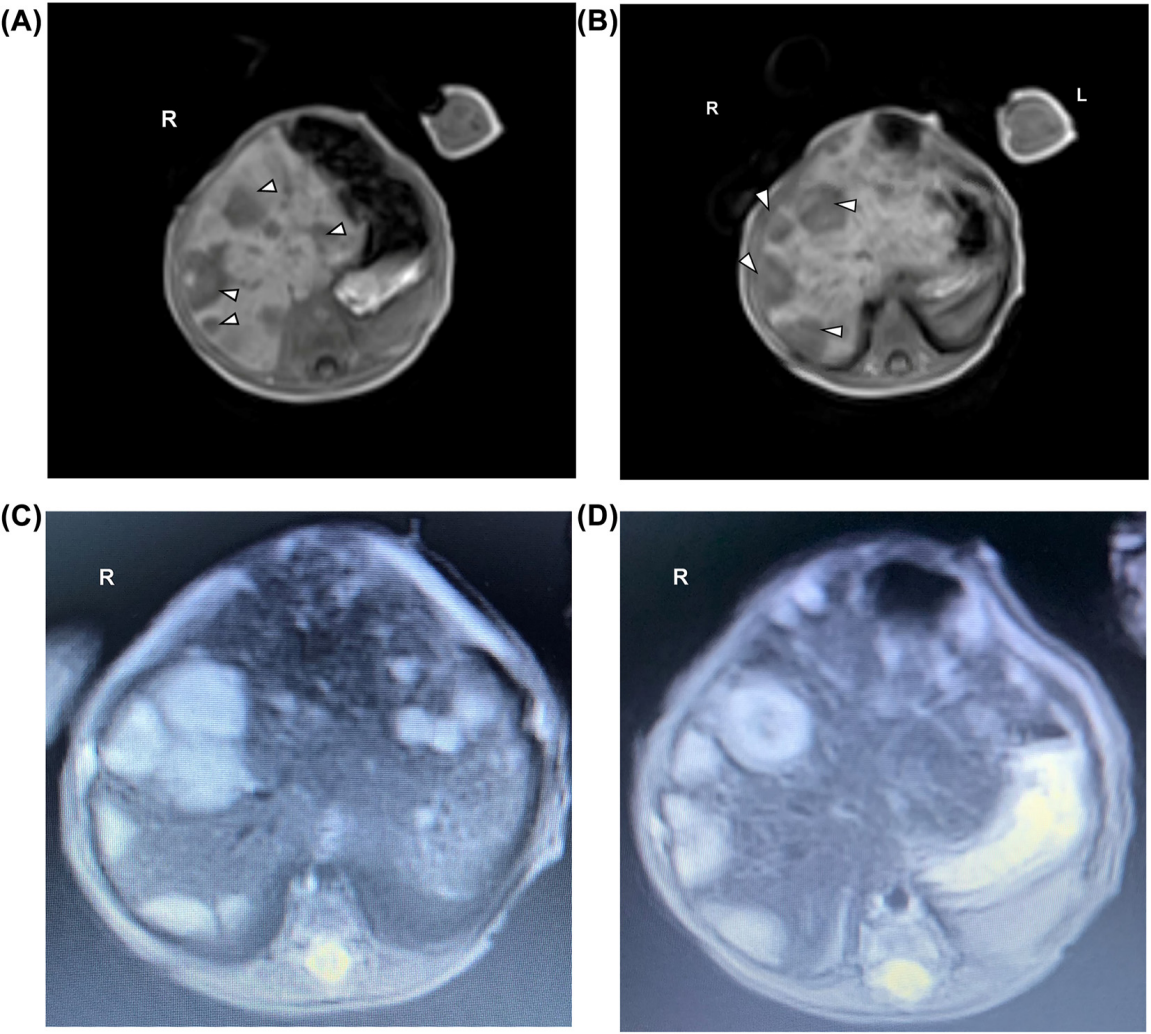
Coronal T1-weighted magnetic resonance imaging (MRI). (A, B) Coronal T1-weighted magnetic resonance imaging (MRI) scan post intravenous contrast agent shows diffusely, heterogeneous enhancement through the liver with distinctly nodular foci of hypointensity (arrows). (C, D) Coronal T2-weighted magnetic resonance imaging (MRI) scan shows diffusely, heterogeneous appearing liver with distinctly nodular foci of hyperintensity (arrows).

A preterm male neonate was born at 32 weeks gestation to a 26-year-old Gravida 3 Para 2 by vaginal delivery. Apgar scores were 2 and 6 at one and 5 min respectively. Pregnancy was complicated with preterm labor. The family history was negative for congenital anomalies, and there was no history of *in-utero* exposure to any known teratogens. Physical examination revealed a weight of 1835 g (50th centile), length of 45 cm (5th centile), and head circumference of 30 cm (25th centile). The infant developed respiratory distress syndrome, and surfactant was administered via an endotracheal tube soon after birth. His respiratory illness was uncomplicated. He required only non-invasive respiratory support for 4 days. Enteral feeding was begun on the 2nd day of life (DOL) but was not well tolerated. Bilious gastric aspirates were noted on the 6th DOL. A contrast study of the stomach and small bowel was reported unremarkable. Antibiotics were begun, and enteral feeding was held for 7 days. Enteral feeding was resumed at 2 weeks of life and was well tolerated. On the 2nd DOL, the infant’s total bilirubin was 3.9 mg/dL and the direct bilirubin was 0.3 mg/dL. The peak serum total bilirubin was 9 mg/dL on DOL 7. On the 24th DOL, a liver function test was obtained to assess the infant’s nutritional status. The total serum bilirubin was 3.9 mg/dL, direct serum bilirubin was 2.8 mg/dL and serum alkaline phosphatase was 1,064 international units per liter (IU/L) Ultrasound of the liver revealed multiple echogenic lesions throughout the liver, the largest lesion measuring about 1.7 × 2.1 x 1.8 cm was located in the right liver (Figure 1). An abdominal MRI was performed, which showed multiple T2 hyperintense and T1 hypodense lesions throughout the liver (Figure 2A–D). The adrenal glands were normal. The working diagnosis was a primary hepatic tumor. A week later (DOL 31), the total and direct bilirubin were 11 mg/dl and 8 mg/dL, respectively, and alkaline phosphatase was 971 IU/L. The PT and PTT were normal. The alpha-fetoprotein was 404,000 ng/mL, urine VMA 125 mg/24 h and urine HVA was 75 mg/24 h. A whole-body MRI found lesions only in the liver. The infant underwent a liver biopsy on DOL 40. The results revealed a metastatic poorly differentiated neuroblastoma [International Neuroblastoma Risk Group (INRG) stage MS: favorable histology neuroblastoma]. Solid tumor panel confirmed favorable prognosis as suspected on prior findings. His respiratory status worsened, and liver function became more abnormal. He developed hypoglycemia requiring IV glucose supplementation from DOL 50 to DOL 58. Chemotherapy (carboplatin and etoposide) was begun on DOL 67 because of respiratory and hepatic dysfunction. The infant developed neutropenia after the chemotherapy treatment, and G-CSF was administered for 10 days. He remained hospitalized for an additional 40 days after chemotherapy for feeding, hypoglycemia, and respiratory issues. The hypoglycemia was attributed to extensive liver metastasis leading to low glycogen storage and hyperinsulinism. Meta-Iodobenzylguanine (IBG) scan with Single Photon Computed Tomography (SPECT/CT) was performed on DOL 93. There was no evidence of MIBG avid disease. Repeat MRI of the liver showed marked resolution of liver lesions as compared to imaging from DOL 44, that is, before the chemotherapy. He was discharged at 5 months of age.

## Discussion

The five stages of international neuroblastoma staging system (INSS) was established more than 30 years ago [5]. They are determined by the location of the tumor at the time of diagnosis. Stage 1: localized tumor with complete gross excision; Stage 2A: localized tumor with incomplete gross excision; Stage 2B: localized tumor with or without complete gross excision, with ipsilateral lymphnodes positive for tumor; Stage 3: Unresectable unilateral tumor invading across the midline, with or without involvement of regional lymph nodes; Stage 4: Any primary tumor that has spread to the distal lymph nodes, bone, bone marrow, liver, skin, and/or other organs; Stage 4S: localized tumor in an infant less than 1 year of age with spread limited to liver, skin, and/or bone marrow [6, 7].

In 2004, the international task force developed a new International Neuroblastoma Risk Group (INRG) Staging System (INRGSS) that was designed to stratify patients at the time of diagnosis before any treatment was started for the NB. The criteria for INRG staging include age, histologic category, grade of tumor differentiation, MYCN status, presence/absence of 11q abnormalities, and tumor cell ploidy [7]. The INRG stages are: L1/L2, L1, L2, M, and MS. Stage L1, localized tumor confined to one body compartment and with absence of image-defined risk factors (IDRFs); Stage L2, locoregional tumor with presence of one or more IDRFs; Stage M, distant metastatic disease (except Stage MS); Stage MS (analogous to INSS stage 4s), metastatic disease confined to skin, liver and/or bone marrow in children less than 18 months of age [5]. Our patient was Stage 4s by the INSS staging system, whereas as per the INRGSS, he was Stage MS at the time of diagnosis.

Approximately 40% of NB patients are under the age of one, 35% are between the ages of one and two, and 25% are older than two years. Our case has Stage MS, which accounts for around 10% of all diagnosed NB cases [7, 8]. The high likelihood of spontaneous regression and generally excellent prognosis, are the hallmarks of Stage MS [9].

Pepper syndrome is described as primary adrenal NB with extensive liver metastases [7], and it refers to stage 4S NBs. However, our patient’s adrenal glands were normal. The staging of the NB in our case suggests that it is a variant of Stage 4S. The presentation initially lead us to consider primary hepatic tumors (hemangioma, hepatoblastoma) instead of NB. Even after the radiology images were obtained, the diagnosis was not clear.

Congenital NBs may be diagnosed on fetal ultrasonography between 33–36 weeks of gestation [4, 10]. Most NBs are found incidentally without maternal or fetal symptoms. But maternal symptoms of catecholamine-related effects e,g, sweating, flushing, and vomiting may be present. Fetal hydrops may occur in the advanced metastatic stage. Neonatal presentation of NB includes abdominal mass/distension, organomegaly, respiratory distress, and sympathetic/catecholamine symptoms (e.g. tachycardia, hypertension). It is important to note that in some cases with congenital NBs, if the NB is not detected antenatally, then the neonate may be asymptomatic and could have a tumor that undergoes spontaneous regression without having been clinically evident. In our case, there was no abdominal distension and no significant hepatomegaly. The ultrasonography was obtained for the evaluation of cholestasis, and such an instance has not been reported previously.

The management of stage MS NB remains inconclusive. Although MS illness without *MYCN* amplification has a favorable prognosis in infants under the age of 12 months, the presence of stage MS NB in a newborn who is less than 2 months old is associated with a bad prognosis, regardless of the *MYCN* copy status, because of the complications [11].

In summary, we report a case of a preterm neonate with cholestasis which lead to the diagnosis of a rare stage MS neuroblastoma.
